# Deterministic Factors Determine the Comammox Community Composition in the Pearl River Estuary Ecosystem

**DOI:** 10.1128/spectrum.01016-22

**Published:** 2022-08-01

**Authors:** Zongbao Liu, Qiaoyan Wei, Dayu Zou, Siyu Zhang, Chuanlun Zhang, Zhexue Quan, Meng Li

**Affiliations:** a Archaeal Biology Center, Institute for Advanced Study, Shenzhen University, Shenzhen, Guangdong, China; b Shenzhen Key Laboratory of Marine Microbiome Engineering, Institute for Advanced Study, Shenzhen University, Shenzhen, Guangdong, China; c School of Life and Environmental Science, Guilin University of Electronic Technology, Guilin, Guangxi, P.R. China; d Shenzhen Key Laboratory of Marine Archaea Geo-Omics, Southern University of Science and Technology, Shenzhen, Guangdong, P.R. China; e Ministry of Education Key Laboratory for Biodiversity Science and Ecological Engineering, Institute of Biodiversity Science, School of Life Sciences, Fudan University, Shanghai, P.R. China; Huazhong Agricultural University

**Keywords:** estuarine ecosystem, comammox, diversity, deterministic process, salinity

## Abstract

Complete ammonia oxidizers (comammox) have been widely detected in riverine and estuarine ecosystems. However, knowledge about the process of comammox community assembly from freshwater to marine environments is still limited. Here, based on deep sequencing, we investigated the community composition of comammox along a salinity gradient in the Pearl River Estuary (PRE), South China. Our results showed that comammox microorganisms in the PRE sediments were extremely diverse and displayed distinct distributional patterns between upstream and downstream habitats. Quantitative PCR demonstrated that comammox was the dominant ammonia-oxidizing microorganism (AOM) in the PRE upstream sediments, and ammonia-oxidizing archaea (AOA) dominated the PRE downstream sediments, while ammonia-oxidizing bacteria (AOB) were not dominant in any section of the PRE. Neutral modeling revealed that stochastic processes explained a limited part of the variation in the comammox community. The majority of beta nearest-taxon index values were higher than 2, indicating that comammox community assembly in the PRE sediments was better explained through a deterministic process than through a stochastic process. Salinity and total nitrogen were the most important contributing factors that shaped the comammox community. This study expanded the current knowledge of the diversity and niche preference of comammox in the estuarine ecosystem, and further enhances our understanding of the assembly of comammox community from freshwater to marine environments.

**IMPORTANCE** Microbial communities are shaped by stochastic (emigration, immigration, birth, death, and genetic drift of species) and deterministic (e.g., environmental factors) processes. However, it remains unknown as to which type of process is more important in influencing the comammox community assembly from freshwater to marine environments. In this study, we compared the relative importance of stochastic and deterministic processes in shaping the assembly of the comammox community, which demonstrated that the deterministic process was more important in determining the community assembly patterns in the PRE ecosystem.

## INTRODUCTION

Nitrification, the microbial oxidation of ammonia to nitrate via nitrite, plays a crucial role in maintaining the balance of global nitrogen cycling. Over the past century, traditional nitrification processes have been generally assumed to be a two-step process involving ammonia-oxidizing archaea (AOA) (1) or ammonia-oxidizing bacteria (AOB), which first transform ammonia to nitrite, followed by nitrite oxidation by nitrite-oxidizing bacteria (NOB) (2). However, the recent discovery of single microorganisms performing complete oxidation of ammonia to nitrate (complete ammonia oxidizers, comammox) in the *Nitrospira* genus has greatly challenged the 100-year-old concept of two-step nitrification (3, 4). According to the results of recent studies, comammox communities have been detected in a wide range of environments, including freshwater (5), tidal zone sediments (6), activated sludge (7), forest and paddy field soils (8), and rice rhizospheres (9), with possible important contributions to the oxidation of ammonia and nitrite on a regional to global scale. Intriguingly, to date, there is no evidence to support the presence of comammox bacteria in marine environments. However, recent evidence suggests the presence of comammox in estuarine tidal flat wetlands, the key transition zone of land and marine interaction (10–12). Yet, the process of comammox community assembly from land to marine environments remains poorly understood.

Estuarine ecosystems link of land, freshwater, and ocean, tend to exhibit extreme dynamics due to seasonal and tidal activity, and are hot spots for nitrogen biogeochemical cycling, including the nitrification process (13, 14). While diverse comammox microorganisms have been found in riverine and estuarine seawater environments, they have not yet been identified in open ocean water or sediment samples (3, 4). Thus, it is reasonable to speculate that the comammox communities inhabiting estuarine environments are likely relocated from terrestrial ecosystems through migration. It would the follow that stochastic processes (emigration, immigration, birth, death, and genetic drift of species) play an important role in structuring the comammox community in estuarine ecosystems. However, the role of stochastic processes in assembling the comammox community in natural ecosystems is barely known. Meanwhile, there are also many examples of deterministic factors (e.g., salinity, pH, and nutrient concentrations) that strongly influence comammox community composition in estuarine ecosystems (10, 11, 13, 15). However, it remains unknown as to which type of process, stochastic or deterministic, is more important in influencing the process of comammox community assembly from freshwater to marine environments. Such knowledge is required for a comprehensive understanding of the community assembly of estuarine comammox microorganisms.

The Pearl River is the second largest river in China. The Pearl River Estuary (PRE) continuously receives large flows of water and sediment from the river, and is also affected by the intrusion of saltwater downstream of the open ocean. In addition, since the PRE is rich in nutrients from the wastewater of surrounding cities (such as Guangzhou, Foshan, and Shenzhen) and industries, it has been confirmed as a hot spot of microbial nitrification (16–18). Related studies in estuaries showed that comammox communities were widely distributed in estuarine habitats with different salinity gradients (5, 13, 15). Meanwhile, numerous studies have reported that changes in nutrient concentrations have a strong influence on microbial communities in the PRE, including archaeal and bacterial communities (19–23). As such, the PRE, is an ideal area to study the process of comammox community assembly from freshwater to marine environments. In this study, we aimed to (i) comprehensively explore the diversity and phylogeny of comammox communities in the PRE ecosystem based on *Nitrospira amoA* genes through deep high-throughput sequencing, (ii) investigate the abundance of comammox in the study area, and compare it with that of AOA and AOB using real-time quantitative PCR (qPCR) analysis, and (iii) evaluate the relative importance of stochastic and deterministic processes in shaping the assembly of comammox community.

## RESULTS

### Physicochemical characteristics of the sediment samples.

The physicochemical properties of the sediment samples used in this study are summarized in Table S2. From upstream to downstream, salinity showed a significant difference from 0.2 to 39 practical salinity units (psu), and the lowest and the highest salinity were observed at DSR3 (0.2 psu) and PRE9 (39 psu), respectively. In addition to sampling site, salinity also varied by sampling season. At sites SZB1, SZB5 and SZB10, salinity was significantly different between the adequate rainfall in July 2016 (wet season) and the shortage of rainfall and water in February 2017 (dry season). Regarding the other parameters, nutrient concentrations of ammonium nitrate (NH_4_^+^), total organic carbon (TOC) and total carbon (TC) were generally higher in the wet season than those of the dry season. The results may indicate that the biogeochemistry of the PRE varied as a function of rainfall and water. Furthermore, regarding the other parameters, NH_4_^+^, total nitrogen (TN), and TOC concentrations at sites with relatively low salinity (≤10 psu) were obviously higher than those at the other sites. Notably, the concentration of NH_4_^+^ in DSR3 sediment was much higher than that in the other samples, up to 423.77 mgN/kg (sediment dry weight). Conversely, relatively stable pH values were found among the sediment samples (ranging from 6.98 to 7.92). Moreover, pairwise comparisons of environmental factors revealed that NH_4_^+^, TOC, and TC were significantly correlated with salinity (*P < *0.001, Pearson correlation), and all displayed the same trend across different sites. Further, based on the salinity level and the sampling site distribution, the sampling sites were separated into three regions, defined as low (≤10 psu), middle (10–30 psu), and high (≥30 psu) salinity areas, respectively (Table S2 and Fig. S1).

### Alpha and beta diversity of the comammox community.

To determine the diversity of comammox bacteria, deep high-throughput sequencing was conducted using the comammox-specific primer sets A189Y/C576r (for first-round PCR) and CA209f/C576r (for second round PCR) (24). Overall, 1,868,039 sequences were retrieved from the 22 sediment samples after trimming and chimera removal, with 62,848–104,277 sequences per sample. Further, 740 and 101 unique OTUs were identified based on 95% and 90% amino acid sequence similarity cutoff thresholds, respectively. Table 1 shows the alpha diversity properties of comammox. At a 95% clustering threshold, the number of operational taxonomic units (OTU) ranged from 49 (PRE11) to 559 (PRE2); at a 90% clustering threshold, the number of OTU ranged from 11 (PRE11) to 89 (PRE2). Alpha diversity, as measured by the Chao1, Shannon and Simpson indices, showed an obvious decreasing trend from upstream to downstream of the PRE. The Chao1 value in sediments with low salinity levels (≤10 psu) was significantly higher than that in high salinity level sediments (≥30 psu) (*P < *0.01) (Fig. S2). Furthermore, the rarefaction curves computed from the observed OTUs nearly reached the plateau phase at the sequence level of 62,848 in all samples, and the accumulation curves reached the saturation phase (Fig. S3), indicating that the current numbers of sequences and sample sets were sufficient to capture the diversity of comammox in the PRE sediments.

**TABLE 1:** Diversity indices with 95% or 90% amino acid identity cutoffs for comammox *amoA* gene sequences^*a*^

Samples^*b*^	Qualified reads	95% cut-off				90% cut-off			
		OTUs	Chao1	Shannon	Simpson	OTUs	Chao1	Shannon	Simpson
DSR3_wet	71319	294	404	2.28	0.82	62	76	2.11	0.81
DSR6_wet	79504	311	385	2.33	0.77	70	82	1.71	0.63
SZB1_wet	73535	245	318	1.49	0.50	61	64	1.36	0.47
SZB5_wet	102167	270	356	1.22	0.38	70	75	1.10	0.43
SZB10_wet	87719	261	354	1.45	0.46	61	65	1.35	0.55
DSR3_dry	62848	295	372	1.95	0.72	64	66	1.67	0.67
DSR6_dry	68518	346	407	2.51	0.77	68	73	2.10	0.75
SZB1_dry	82681	353	449	2.63	0.85	80	95	2.11	0.82
SZB5_dry	99182	328	388	2.00	0.67	70	75	1.67	0.68
SZB10_dry	87540	359	426	2.59	0.83	71	73	2.16	0.80
PRE1	70053	493	543	3.51	0.91	82	88	2.47	0.86
PRE2	97298	534	619	3.53	0.92	89	92	2.65	0.88
PRE3	81543	559	609	3.48	0.92	87	93	2.62	0.89
PRE4	80439	395	450	2.79	0.85	83	84	2.32	0.84
PRE5	92978	401	511	2.81	0.86	79	86	2.20	0.81
PRE6	102419	341	424	2.16	0.69	73	96	1.39	0.56
PRE7	95291	173	206	1.61	0.67	49	54	1.15	0.57
PRE8	78863	343	415	2.66	0.83	68	79	2.31	0.81
PRE9	89549	357	424	2.84	0.86	70	72	2.30	0.83
PRE10	104277	367	493	2.16	0.66	71	77	1.39	0.50
PRE11	77345	49	61	0.49	0.17	11	13	0.25	0.11
PRE12	82971	51	62	0.45	0.15	11	17	0.26	0.11

a All the samples were rarified to 62,848 reads per sample.

b DSR, Dasha river; SZB, Shenzhen Bay; PRE, Pearl River estuary; wet, wet season; dry, dry season.

**FIG 1:**
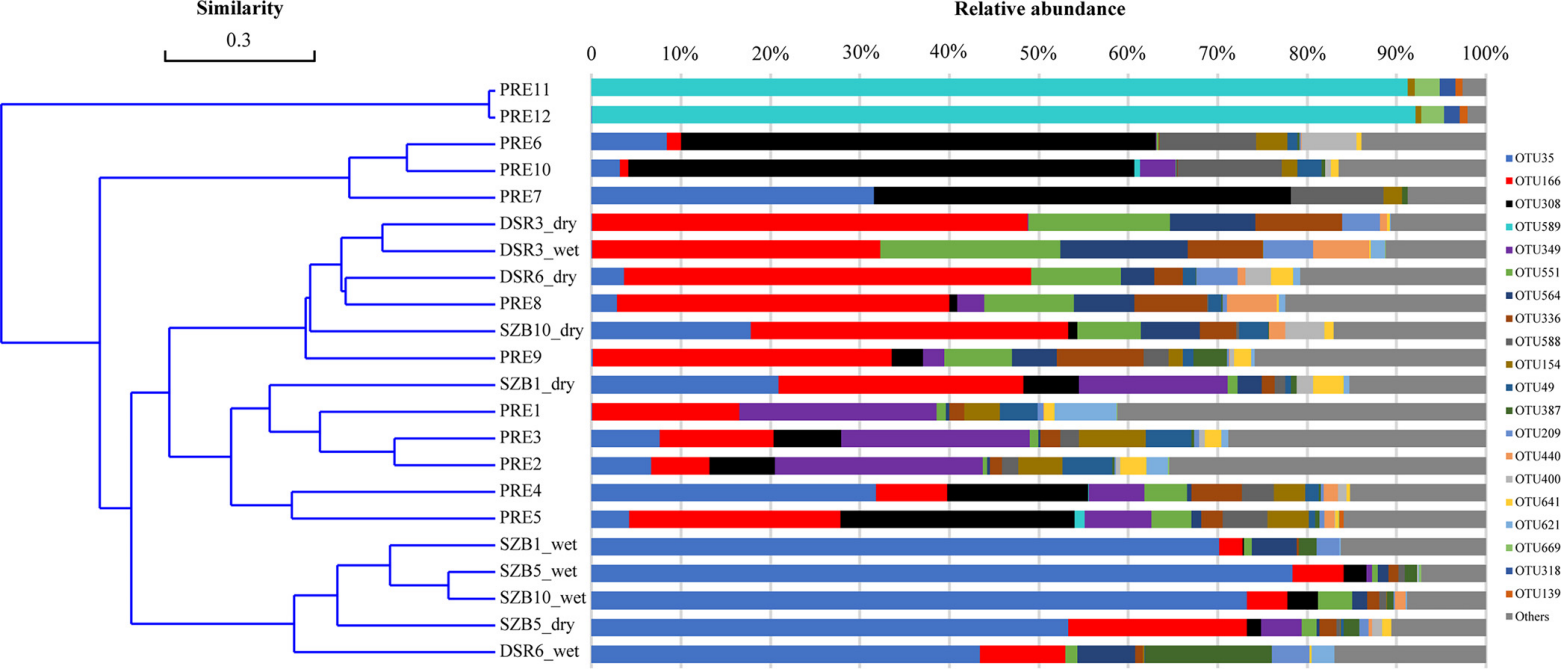
Community compositions of comammox in the 22 the PRE sediments. Comammox *amoA* sequences were clustered into operational taxonomic units (OTUs) at 95% similarity cutoff thresholds; Sample clustering was performed by using the unweighted pair-group method with arithmetic means (UPGMA) based on Bray-Curtis distance. PRE, Pearl River estuary; DSR, Dasha river; SZB, Shenzhen Bay; wet, wet season; dry: dry season.

The comammox community composition of the 22 sediment samples from the PRE region is shown in Fig. 1. Based on 95% similarity cutoff thresholds, the top 13 dominant OTUs (with a proportion higher than 1%) accounted for 79.5% of the total OTU abundance. However, sample clustering analysis with the UPGMA tree showed that there was still distinct OTU compositions between the different sampling sites (Fig. 1). Similar to the UPGMA tree analysis, the clustering analysis with heatmap visualization showed significant differences in the OTU distribution among sediments from different sampling sites (Fig. S4). The differences in the community composition of comammox among different sampling sites were further analyzed by nonmetric multidimensional scaling (NMDS) analysis based on Bray-Curtis distance matrices. The results revealed that sampling sites had a significant influence on comammox community composition (PERMANOVA, R^2^ = 0.372, *P < *0.01) (Fig. S5).

### Phylogenetic analysis of the comammox *amoA* genes.

A phylogenetic tree of the 20 dominant comammox *amoA* OTUs (with a proportion higher than 0.5%) was constructed using the maximum composite likelihood method (Fig. S6). Consistent with previous results, the OTU sequences were branched into two clades, where 19 out of the 20 OTUs were affiliated with clade A and accounted for approximately 83.9% of the total comammox *amoA* gene sequences. The clade A comammox sequences were further divided into two monophyletic groups: clades A.1 and A.2. Among clade A.1, OTU35, OTU166, and OTU308 clustered with *Nitrospira* sp. WS110, *Candidatus* Nitrospira nitrosa and *Candidatus* Nitrospira nitrificans, respectively, were the top 3 dominant comammox *amoA* OTUs in the PRE sediments. Eight OTUs affiliated to comammox clade A.2, in which OTU589 clustered with metagenome-assembled genomes (MAGs) *Nitrospira* sp. SU_4_4 and RL_2_10 was the most abundant OTU. Only one representative OTU was affiliated with clade B and accounted for 0.78% of the total *amoA* sequences (Fig. S6).

**FIG 2:**
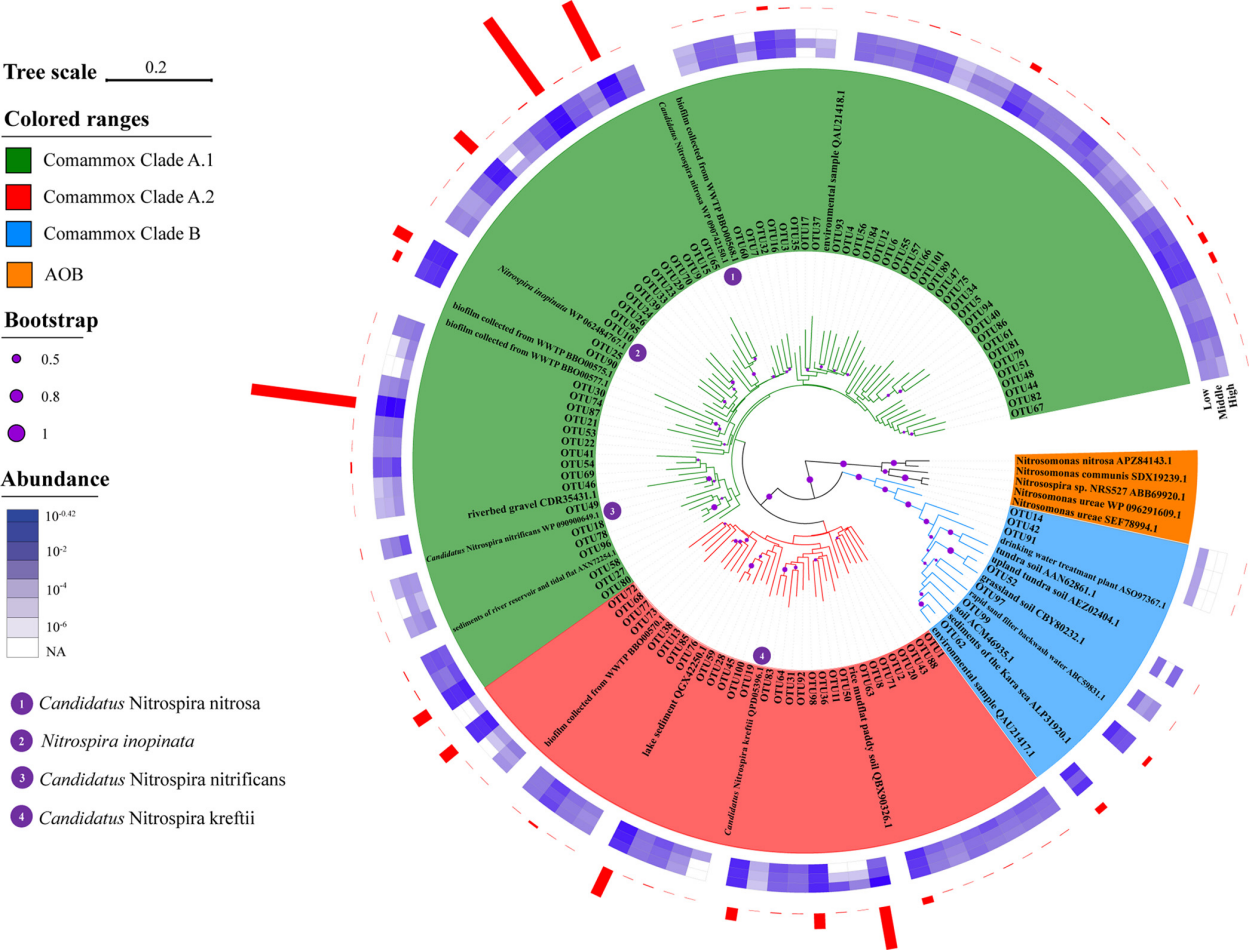
Phylogenetic analysis of representative comammox *amoA* genes obtained in this study. Neighbor-joining phylogenetic tree included 101 OTUs (90% clustering threshold) identified from the 22 PRE sediments, and 3 known cultured comammox *Nitrospira*, 5 AOB and other representative *amoA/pmoA* sequences from NCBI database. The reference sequences in this tree represent comammox *amoA* gene sequences collected from NCBI nr database that clustered at 90% amino acid identity threshold. The color gradient of the heatmap indicates the relative proportion of each OTU in the sediments with different salinity levels. The red bar graph indicates the relative proportion of each OTU in the total PRE sediments.

Another phylogenetic tree comprising 101 OTUs (based on 90% clustering threshold) and reference sequences from the NCBI nr database was constructed to reveal the evolutionary history of comammox in the PRE region (Fig. 2). As expected, the obtained OTUs formed distinct clusters from canonical bacterial *amoA* sequences. Among the 101 OTU sequences, only 7 OTUs were affiliated with clade B, and showed relatively close phylogenetic relationships with comammox *amoA* sequences from various types of environmental samples, such as tundra soil, grassland soil, and drinking water treatment plants (Fig. 2). Comammox in this clade were mainly found in low salinity areas, accounting for approximately 50% of the total sequences of clade B. For the remaining OTUs, sequences clustered with *Candidatus* Nitrospira nitrosa and *Candidatus* Nitrospira inopinata were relatively highly abundant. Remarkably, a portion of OTUs affiliated with clade A.1 formed a very distinct group in the phylogenetic tree without any reference sequences, indicating the presence of unique comammox bacteria in the PRE sediments (Fig. 2).

### Evaluation of factors in structuring the comammox community.

**FIG 3:**
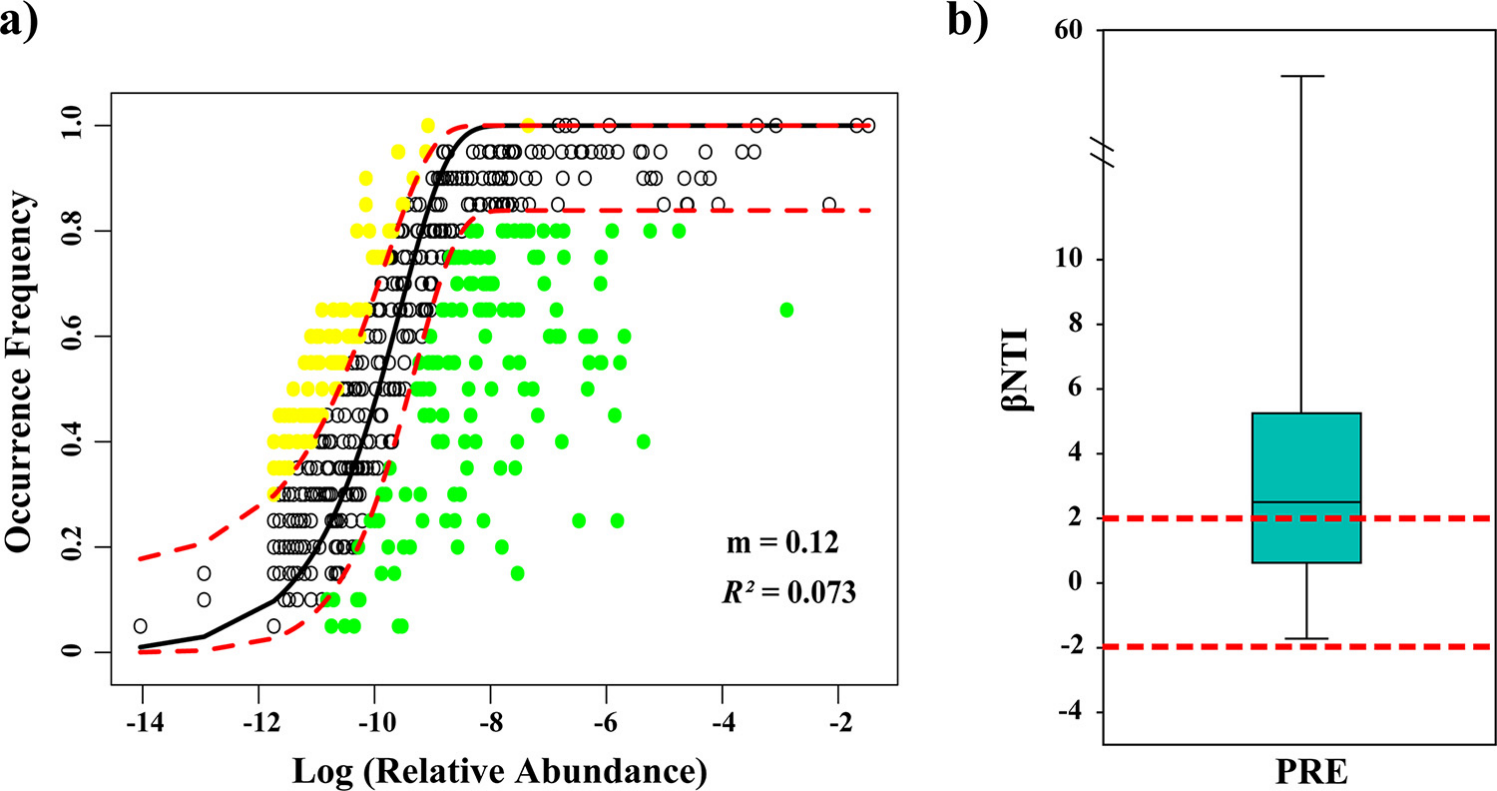
**(**a) Fit of the neutral model for comammox community in the PRE sediments. Yellow and green dots indicate the OTUs that occur more and less frequently than predicted by the model. Dashed blue lines represent 95% confidence intervals around the model prediction (solid black line). *m* indicates the immigration rate of comammox community; *R^2^* indicates the fit to the neutral model. (b) Box plots of beta nearest-taxon index (βNTI) values in the PRE sediments. Horizontal dashed lines (βNTI values of 2 and -2) indicate thresholds of significance.

We used the NCM to assess the potential contribution of stochastic processes to the variation comammox community in the PRE sediment. The results showed that this model did not fit well the comammox community assembly in the PRE sediments (*R^2^* = 0.073, m = 0.12) (Fig. 3a). Further, βNTI was performed to evaluate the relative importance of stochastic and deterministic processes in shaping comammox community assembly. As shown in Fig. 3b, the majority of the βNTI values among the samples were higher than 2, suggesting that a deterministic process (e.g., environmental filtering) played a more important role in the PRE sediment comammox community assembly.

**FIG 4:**
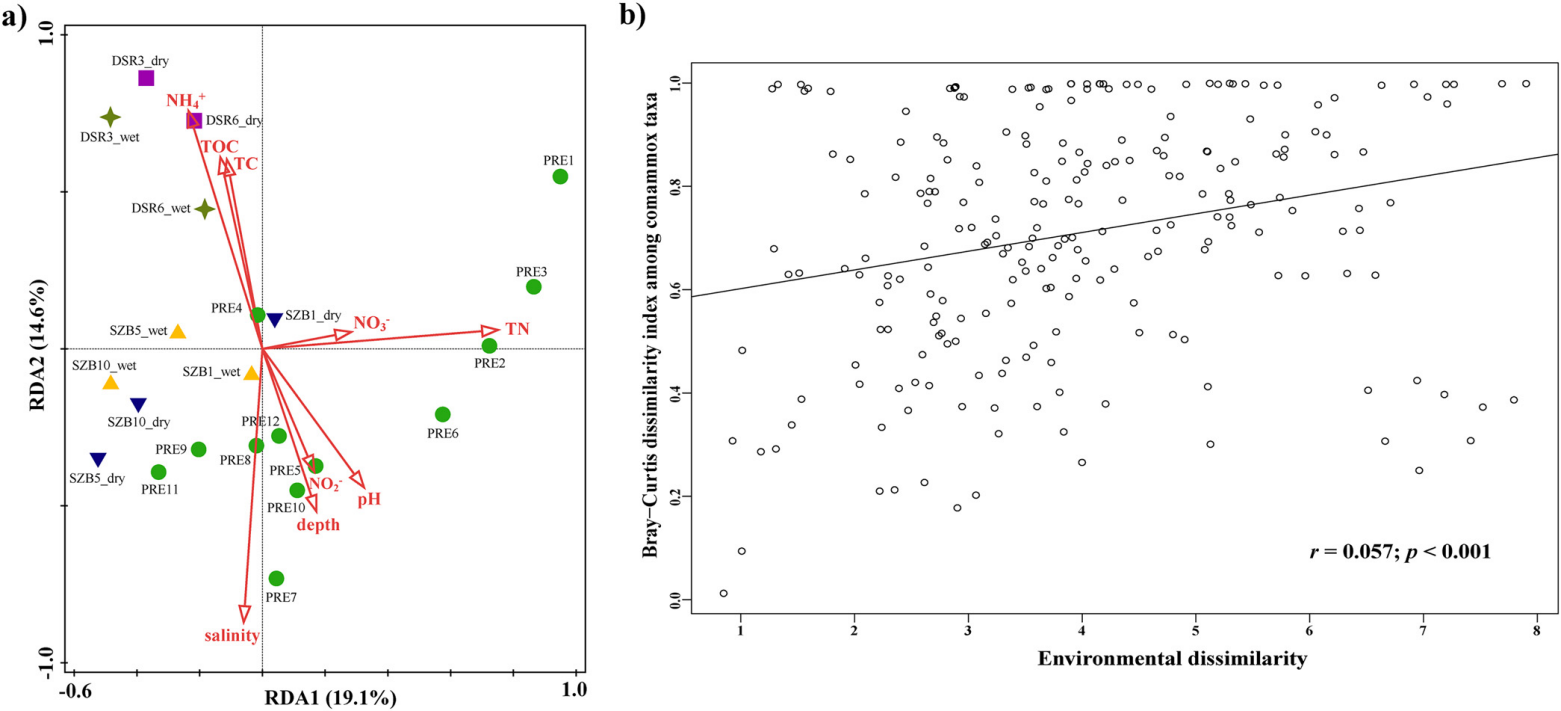
(a) Redundancy analysis (RDA) for the relationship between comammox community composition with the environmental parameters. Symbols in different colors indicate samples from different sampling areas in the PRE. The values of RDA1 and 2 are percentages that the corresponding axis can explain. (b) Relationship between comammox community dissimilarity (Bray-Curtis distance) and environmental dissimilarity.

Redundancy Analysis (RDA) was next performed to reveal the relationships between comammox communities and environmental factors. The environmental variables in the 2 axes of RDA explained 19.1% and 14.6% of the variance of the comammox community, respectively (Fig. 4a). In detail, salinity, TN, and NH_4_^+^ were the top 3 important contributing factors that shaped the comammox community composition, explaining 12.7%, 12.5%, and 9.7% of the total community variation, respectively (*P < *0.01) (Table S3). In addition to the above 3 factors, depth also significantly explained the variation in *amoA*-based comammox community, explaining 7.9% of the total variances (*P < *0.05) (Table S3). Furthermore, comammox community dissimilarity increased significantly with environmental dissimilarity (Fig. 4b) (Mantel test; *r *= 0.057, *P < *0.001), which further confirmed the important roles of environmental factors in the assembly of the PRE sediment comammox community. In addition, a total of 39.8% of the total variances remained unexplained by these environmental factors.

### Abundances of comammox, AOA, and AOB.

The abundance of comammox clade A *amoA* gene at 17 sites in the PRE region was determined by the qPCR analysis. For comparison, the canonical bacterial and archaeal *amoA* genes were also quantified. Considering the differences in sediment physical properties in this study (grain size, humidity, etc.), which could significantly influence the rate of microbial DNA extraction, the abundance of ammonia oxidizers in each sediment was normalized by the total number of 16S rRNA genes instead of by the weight. Obtained from the high-throughput sequencing analysis, the proportion of clade B among the total comammox population in each of the PRE sediments was shown in Fig. S7. The abundance of clade B comammox population was therefore calculated from the ratio of clade A to clade B. After combining the 2 clade comammox populations, the abundance of comammox *amoA* genes in the 22 sediment samples ranged from 2.53 × 10^−4^ to 1.15 × 10^−2^ copies per copy of 16S rRNA gene (Fig. S8). As salinity increased, the relative abundance of comammox decreased in samples from the PRE upstream to downstream. In contrast, the relative abundance of AOA increased obviously along the Estuary, ranging from 2.48 × 10^−5^ to 1.77 × 10^−2^ copies per copy of the 16S rRNA gene. The variations of the relative abundance of AOB among the sediment samples were less obvious than those of AOA and comammox. Furthermore, the *nxrB* gene abundance of NOB showed similar trends to comammox, ranging from 5.79 × 10^−4^ to 3.08 × 10^−2^ copies per copy of the 16S rRNA gene (Fig. S8).

**FIG 5:**
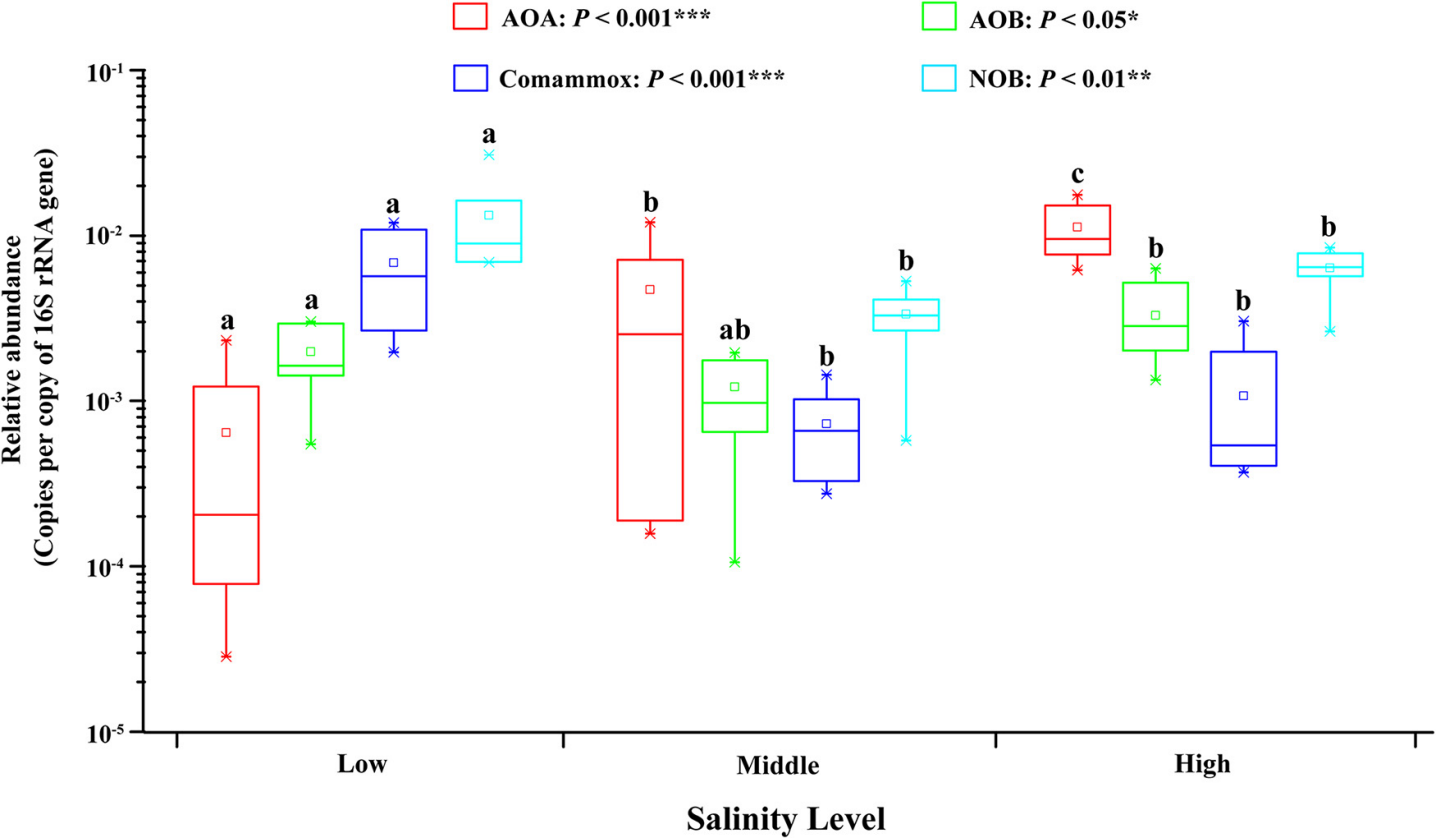
Relative abundances of AOA, AOB, comammox and NOB in the PRE sediments with different salinity levels. The red, green, blue, and cyan boxes represent AOA, AOB, comammox and NOB, respectively. The lower case letters (a) and (b) indicate significant differences among different sites (one-way ANOVA; Duncan test; *P* < 0.05).

We further compared the relative abundance of AOA, AOB, and comammox in sediments of different salinity levels. Among the 22 sediments, comammox was dominant in samples with low salinity, and AOA was dominant in samples with middle and high salinity, while AOB were not dominant at any salinity level of the analyzed samples. In low salinity area sediments, the average abundance of comammox was 9.9 and 3.3 times that of AOA and AOB (Fig. 5), suggesting the important role of comammox microorganisms in ammonia oxidation in this area. Whereas, the relative abundance of comammox in the middle and high salinity areas was significantly lower than that of AOA and AOB (one-way ANOVA, *P < *0.05). Overall, the relative abundance of archaeal and bacterial *amoA* genes varied significantly in different groups clustered by salinity level (*P < *0.05) (Fig. 5).

### Influence of environmental factors on the abundance of ammonia oxidizers.

**FIG 6:**
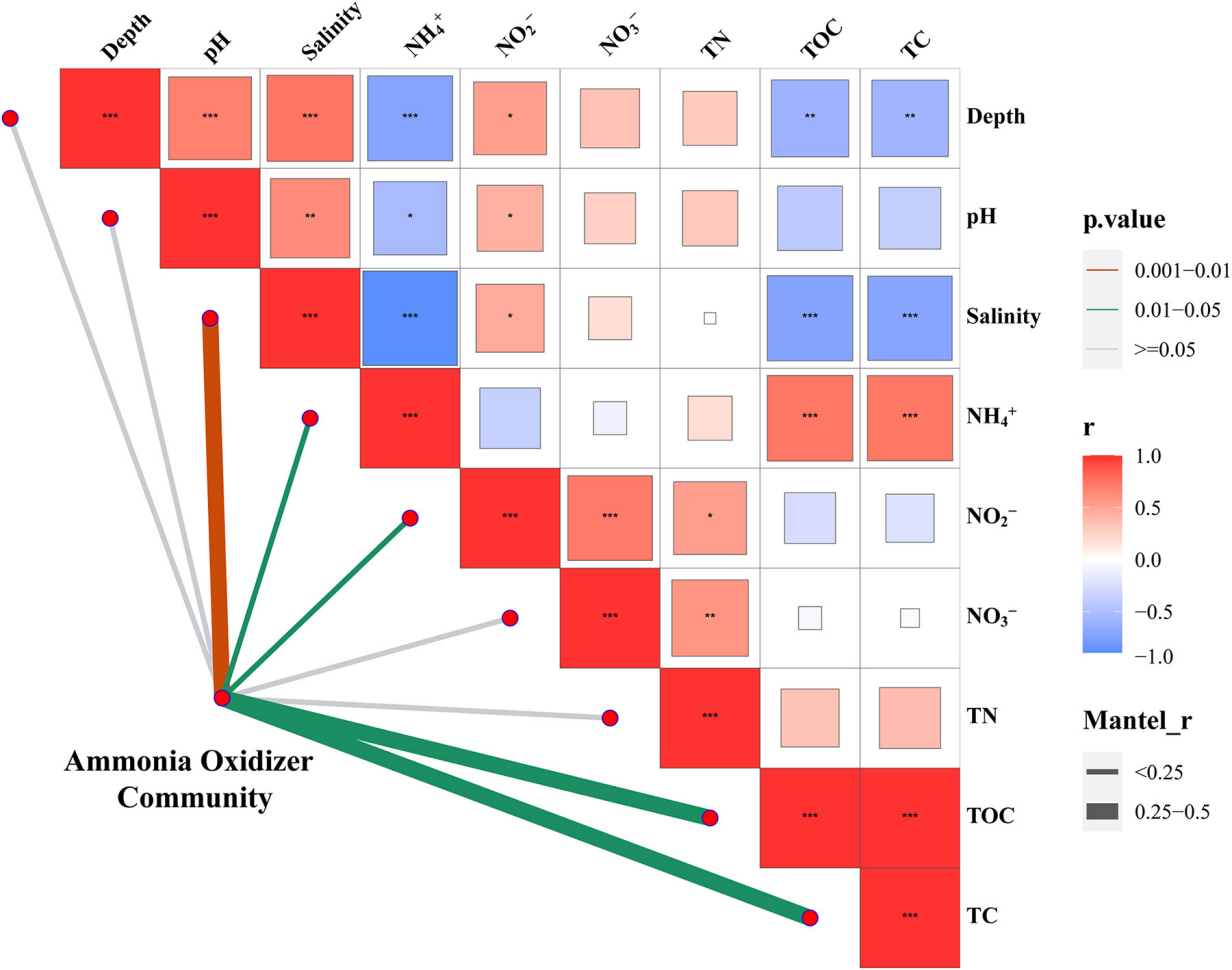
Pairwise comparisons of environmental factors and their effects on the community composition of ammonia oxidizers. The color gradient indicates Spearman’s correlation coefficient. Edge width corresponds to the Mantel’s r statistic for the corresponding distance correlations, and edge color represents the statistical significance based on 9,999 permutations.

The Mantel test results, which evaluated how the abundance of AOA, AOB, and comammox *amoA* genes were constrained by environmental factors, revealed that the composition of the ammonia-oxidizing communities was closely correlated with multiple sediment attributes, and exhibited the most significant correlation with salinity, followed by TOC, TC, NH_4_^+^ and nitrite nitrogen (NO_2_^−^) (Fig. 6). Pearson correlation analysis showed that the relative abundance of comammox was negatively correlated with salinity, pH value and sediment depth, and positively correlated with TN, TOC, TC, NH_4_^+^, nitrate nitrogen (NO_3_^−^), and NO_2_^−^ concentrations. Unlike the comammox, AOB abundance was negatively correlated with TOC, TC and NH_4_^+^ concentrations, and positive correlated with salinity (Fig. S9). Seven out of 9 environmental factors were significantly correlated with AOA abundance. Among them, TOC, TC, and NH_4_^+^ concentrations were negatively correlated with AOA abundance, while salinity, pH value, sediment depth, and NO_2_- showed positive correlations with their relative abundance (Fig. S9). In particular, the relative abundances of AOA, AOB, and comammox were all significantly correlated with salinity, indicating the important role of this factor in affecting the distribution of ammonia-oxidizing communities in the PRE sediments.

## DISCUSSION

### Comammox in the PRE sediments exhibited high diversity.

Since the discovery of comammox, the diversity and distribution of this community have been investigated in various natural and engineered ecosystems (6, 7, 13, 24–26). To comprehensively explore the diversity of the comammox community in the PRE ecosystem, we performed the deep high-throughput sequencing of the comammox *amoA* gene using a universal PCR primer set targeting both clade A and clade B. Remarkably, the comammox exhibited extremely high diversity in our investigated the PRE ecosystem, especially in upstream sediments. Based on a 95% clustering threshold, a total of 740 *amoA* gene OTUs were obtained from the 22 sediment samples, with 49–559 OTUs per sample (Table 1), far more than those obtained from other ecosystems. For example, Sun et al. (12) investigated the diversity of comammox *amoA* gene in coastal wetlands surface sediment, based on 95% nucleotide similarity, and only 118 OTUs were obtained from these samples, with the number of OTUs for each sample ranging from 17 to 70. Besides the ecological niche factor, the great distinction in diversity of comammox between the current and other studies could be associated with the use of different primer sets and sequencing depth. Consequently, a universal primer set with high coverage combined with deep sequencing might be necessary to capture a comprehensive insight into the diversity of comammox in natural ecosystems. Furthermore, many OTUs identified from the PRE sediments shared relatively low amino acid sequence identity (<90%) with deposited comammox *amoA* sequences in public databases, indicating that the results of this study expand the current knowledge of the diversity of the comammox community.

### Phylogenetic analysis supports the presence of unique comammox.

Previous studies have suggested that the comammox community displays high phylogenetic diversity (24, 27, 28). In the current study, the phylogeny of *amoA* genes from the PRE comammox community was investigated by constructing a phylogenetic tree with 101 OTUs (90% clustering threshold) and representative reference sequences from the NCBI nr database (Fig. 2). Our findings are consistent with previous phylogenetic analyses in that the OTU sequences were branched into 2 clades (clades A and B), and the clade A sequences were further divided into 2 monophyletic groups: clades A.1 and A.2 (24, 27, 29). Comammox clade B was a small group in the PRE sediment, accounting for only 0.78% of all sequences, and clade A.1 was the dominant cluster and possessed the highest phylogenetic diversity, comprising 73.2% (Fig. 2). The *amoA* genes of comammox in clades A.2 and B were clustered together with reference sequences from various environments and shared high sequence identity with these sequences, which indicates their wide distribution in natural and engineered ecosystems. Clade A.1 contained 3 important culture species: *Nitrospira inopinata*, *Candidatus* Nitrospira nitrosa and *Candidatus* Nitrospira nitrificans (3, 4). Notably, a part of OTUs affiliated to clade A.1 formed a very distinct group in the phylogenetic tree and shared <90% sequence identity with deposited sequences in public databases (Fig. 2). Therefore, it is reasonable to assume that comammox species or strains distinct from those identified in previous studies might coexist in the PRE ecosystem.

### Comammox dominate ammonia oxidizers in the PRE upstream sediments.

Comammox bacteria have been found to co-exist with traditional ammonia oxidizers in various habitats, such as soil (30), mangrove sediments (27), tidal flat sediments (31), and wastewater treatment plants (32), and there has been controversy over which community is more dominant, AOA (22, 27, 33) or comammox (29, 34, 35). In this study, the relative abundance of comammox varied significantly from the upstream to downstream PRE (*P < *0.001) (Fig. 5). The abundance of the comammox *amoA* gene in the PRE upstream sediments was significantly higher than that of the AOA and AOB *amoA* genes (*P < *0.01), accounting for 71.2% of the total ammonia-oxidizing microorganism population. This result might suggest that comammox makes an important or even dominant contribution to ammonia oxidization in the PRE upstream sediments. Further, the identification of the dominant position of comammox in ammonia-oxidizing prokaryotes in the PRE sediments with high ammonium concentrations challenges the commonly accepted view that comammox has a strong affinity for ammonia and is generally more competitive at low ammonium concentrations (3, 4). Moreover, in the middle and downstream of the PRE, the abundance of AOA was significantly higher than that of AOB and comammox (*P < *0.01) (Fig. 5), suggesting that AOA rather than comammox dominated the ammonia oxidation in this area. This finding corresponds well with previous studies that AOA has a strong adaptability to different salinity gradients, and generally has a dominant position in the sediments with high salinity (27, 36, 37). Furthermore, previous studies have proposed that the AOA have an extremely high affinity for ammonia, and they thus have a higher ability to survive in oligotrophic environments (38, 39). Consequently, differences in adapting to salinity and ammonia concentration levels may be an important driver of niche differentiation of ammonia oxidizers in the PRE ecosystem.

In addition, the abundance of comammox bacteria was negatively correlated with AOA and positively correlated with AOB, indicating that there might be a synergistic or competitive relationship between these bacteria. Previous study reported that anaerobic ammonia-oxidizing (anammox) bacteria were also important players in the nitrogen cycle in the PRE, contributing to approximately 17.49% of the total microbial nitrogen loss (19). We hypothesized that the presence of comammox bacteria in the PRE might be negatively correlated with anammox bacteria, as comammox bacteria oxidize ammonia directly to nitrate, which reduces the source of nitrite for anammox bacteria and may have a detrimental effect on the growth of anammox bacteria (3, 4, 40). Thus, the competition-cooperation among the ammonia oxidizers should be another factor contributing to their community structure and dynamics in the PRE ecosystem.

### Deterministic processes play a more important role in shaping comammox community composition.

Stochastic and deterministic processes are two of the most well-documented ecological theories that explain microbial community assembly patterns (41). Various studies have demonstrated that stochastic processes, such as dispersal and drift, significantly influence the assembly of microbial communities in aquatic environments (42–44). However, in the current study, we observed that the NCM did not fit well the comammox community assembly in the PRE sediment (Fig. 3a), suggesting that stochastic processes play a limited role in shaping the assembly of the comammox community. For example, the majority of the βNTI values among the sediment samples were higher than 2 (Fig. 3b), indicating that environmental filtering (deterministic process) overwhelmed spatial-related dispersal (stochastic process) in the PRE comammox community assembly. In estuarine ecosystems, comammox communities are strongly stressed or filtered by large variations in environmental factors. The constant fluctuations in hydrological and environmental factors, such as salinity, pH, dissolved oxygen (DO) and nutrients create harsh conditions in estuaries, where only well-adapted species can thrive (45). Therefore, it is not surprising that environmental filtering was more important than riverine input in driving comammox community assembly in the PRE ecosystem.

The RDA further provided evidence that environmental factors had a strong influence on comammox community diversity and composition, explaining 60.2% of the total community variation. Among the detected environmental factors, salinity was the most important contributing factor in shaping the comammox community, in agreement with previous studies which reported that salinity played a major role in affecting the distribution and community composition of nitrifying microorganisms (13, 27, 46, 47). Salinity changes might directly affect or alter osmoregulation and metabolism of the microbial cell (44), thus could significantly affect the life supporting activities and abundance of different comammox communities. Similar to previous studies in the PRE (45, 47), we observed that nutrient concentrations, such as TN and NH_4_^+^ concentrations in the estuary changed dramatically (Table S2), and their availability contributed substantially to the variation in comammox community composition. Furthermore, biotic factors (e.g., species interactions) also play a role in shaping the microbial communities. As the PRE harbored high diverse microbial community (22, 48, 49), there might exist complex interactions between comammox and other microbial groups, which could be another factor contributing to the distinct distributional patterns of comammox communities in the PRE ecosystems. Regarding which environmental factors influenced the abundance of AOA, AOB and comammox, our study revealed that salinity was most significantly correlated with the composition of the ammonia-oxidizing communities (Fig. 6), indicating that salinity was a key environmental factor that drove the niche differentiation of AOA, AOB, and comammox. For the comammox community, salinity was found to have significant negative correlations with both their diversity and relative abundance. Furthermore, other environmental factors, such as TC, TOC, NH_4_^+^, and NO_2_^−^ were also significantly correlated with the changes in the relative abundance of ammonia-oxidizing communities (Fig. 6). These results provide further support to the conclusion that deterministic processes dominate the comammox community assembly in the PRE ecosystem.

### Conclusion.

Based on high-coverage primers and deep sequencing, this study provides a comprehensive insight into the diversity of comammox in the PRE sediments along a salinity gradient. The results revealed that the diversity and abundance of the comammox community varied significantly from upstream to downstream. Phylogenetic analysis revealed a novel comammox group within clade A that formed a distinct cluster for which no reference sequence existed, implying the existence of potential unique comammox in the PRE ecosystem. In addition, we presented the first effort to compare the relative importance of stochastic and deterministic processes in shaping the assembly of the comammox community, and demonstrated that the deterministic process was more important in determining the community assembly patterns in the PRE sediments. Variations of comammox community were strongly linked to salinity, TN, and NH_4_^+^. The current study provides a comprehensive insight into the assembly of comammox community in estuarine ecosystem. Future studies, including those on the diversity and abundance of comammox linked with the expression activity, culture of comammox harbored in samples with different salinity levels and comparing their genome diversity and environmental adaption, would provide a more profound understanding of the variation in comammox community from freshwater to marine environments.

## MATERIALS AND METHODS

### Sample collection.

In this study, surface sediment samples were collected from 17 sites in the PRE region located on the coast of the South China Sea (Fig. S1). Sample collection was conducted from July 2015 to July 2017. The details of the sample sites and collection periods are provided in previous studies (22, 33). At each site, surface sediment samples were collected with a stainless steel grab sampler, packed in plastic sealed pockets, and immediately placed on ice for transport to the laboratory. Sediment samples were divided into two sets: one set was stored at −40°C prior to DNA extraction, and the other set was stored at 4°C prior to physicochemical analysis.

### Physicochemical analysis.

Salinity and pH were measured in fresh sediments, whereas nutrients were measured in air-dried sediments. The interstitial water of the fresh sediment was collected by centrifugation, and the salinity and pH were measured using an automatic compensation salinity refractometer (ATAGO Co., Japan) and a pH meter (Mettler-Toledo Instruments Co., China). Sediments were air-dried for multiple days until their weight remained unchanged. Each sample was then thoroughly mixed after passing through a 2-mm sieve to remove roots and stones. Sediment ammonium, nitrite and nitrate were extracted from air-dried sediments (10 g) with 100 mL of 1 M KCl by shaking at 180 rpm for 60 min. The concentrations of NH_4_^+^, NO_2_^−^ and NO_3_^−^ were determined using a continuous segmented flow analyzer (SEAL AutoAnalyzer 3 HR, Maquon, WI, USA). A Shimadzu TOC VCPH analyzer (Shimadzu, Japan) was employed to analyze the TN, TC and TOC as reported previously (50).

### DNA extraction and sequencing.

Genomic DNA of the sediment microbial community was extracted from each sediment sample using the DNeasy PowerSoil kit (Qiagen, Germany) following the manufacturer's instructions. The concentration and quality of the extracted total DNA were examined using a NanoDrop-2000c UV-Vis spectrophotometer (ThermoFisher Scientific, USA) and 1% agarose gel electrophoresis. The *amoA* genes of comammox *Nitrospira* were amplified by nested PCR with the primer set A189y (5′-GGNGACTGGGAYTTYTGG-3′)-C576r (5′-GAAGCCCATRTARTCNGCC-3′) and barcoded primer sets CA209f (5′-GAYTGGAARGAYCGNCA-3′)-cob576r on a C1000 Thermal Cycler instrument (Bio-Rad, USA) (24, 51). The amplification reaction and thermal program of the nested PCR were performed as described in our previous studies (27, 51). To increase the specificity of amplification, we employed touchdown PCR to perform the second round of nested PCR. During PCR amplification, negative (i.e., without template DNA) and positive (i.e., recombinant plasmid containing the comammox *amoA* gene) controls were included. The amplified products were confirmed by 2% agarose gel electrophoresis. The purified amplification products were paired-end sequenced (2 × 250 bp) at Novogene Bioinformatics Technology Co., Ltd. (Beijing, China) using an Illumina HiSeq 2500 platform (Illumina, USA).

### Quantification of the *amoA*, *nxrB,* and 16S rRNA genes.

To determine the copy numbers of AOA, AOB, comammox and *Nitrospira* in each sediment, quantitative real-time PCR was performed in triplicate with a LightCycler 96 real-time PCR system (Roche, USA) using the SYBR green qPCR method. The copy numbers of bacterial 16S rRNA genes were also quantified to evaluate the relative abundance of these microorganisms. All qPCRs were carried out in a total volume of 25 μL using the SYBR Premix *Ex Taq* (Tli RNaseH Plus) Kit (TaKaRa, Japan), containing 2 μL of extracted DNA and 0.2 μM each primer. Detailed information regarding the qPCR primers used in this study is summarized in Table S1. Amplifications were performed using the following protocols: 95°C for 3 min, followed by 40 cycles of 15 s at 95°C, 30 s at 55°C (for 16S and AOA), 58°C (for AOB), 57°C (for comammox) and 56°C (for *Nitrospira*) (Table S1), and 45 s at 72°C, followed by a melting curve stage. Standard curves of qPCR were obtained by serially diluting the recombinant plasmids containing the target genes with known copy numbers. Standard curves with an amplification efficiency of 0.90–1.10 and correlation coefficient (*R^2^*) above 0.98 were accepted.

### High-throughput sequencing data processing and analysis.

The raw paired-end reads of each sample were initially pretreated using FastQC (https://www.bioinformatics.babraham.ac.uk/projects/fastqc/) to remove low-quality sequences, and then merged using FLASH (V1.2.7, http://ccb.jhu.edu/software/FLASH/) based on matched overlapping regions. The sequence analysis tool USEARCH (52) was used to check chimeras by comparing with the expanded copper-containing membrane monooxygenase (CuMMO)-related gene database constructed by Wang et al. (51). Subsequently, chimeras were filtered and the high-quality nucleic acid sequences were translated to protein sequences using FrameBot tools in the Functional Gene Pipeline/Repository (http://fungene.cme.msu.edu/) (53, 54). Preprocessed sequences were assigned to operational taxonomic units (OTUs) based on their amino acid sequence similarity (identity = 0.95 and 0.90) by USEARCH using the -cluster_smallmem command. For taxonomic identification of the OTUs, the representative sequences were aligned against the CuMMO-related gene database mentioned above, and the comammox OTU sequences were reconfirmed through a phylogenetic tree constructed with OTU representative sequences and comammox *amoA* reference sequences using MEGA7.0 software with the neighbor-joining method (55). Filtered merged sequences from each sample were then assigned to representative OTUs using the BLASTX algorithm with amino acid identity ≥90% and coverage ≥80%, and OTUs with ≤5 sequences were excluded from downstream analyses. A resampling procedure was applied at a depth of 62,848 sequences for each sample to calculate a variety of diversity indices.

### Statistical analyses.

Sample clustering was performed using Paleontological STatistics (PAST) software (version 3.16) and the unweighted pair-group method with arithmetic means (UPGMA). A variety of alpha diversity indices of comammox bacteria, such as the number of OTUs, richness estimator (Chao1), and diversity indices (Shannon-Weiner) were calculated using the vegan package (56) in the R environment based on OTU thresholds of 95% and 90%. Nonmetric multidimensional scaling (NMDS) analysis based on Bray-Curtis distances was performed in the R vegan package to evaluate the similarity of comammox community compositions across different sites. To analyze the phylogenetic characteristics of the comammox microorganisms obtained in this study, the representative OTUs were aligned with the comammox reference sequences collected from the National Center for Biotechnology Information (NCBI) non-redundant (nr) nucleotide database, and neighbor-joining trees were constructed using MEGA7.0 based on the maximum composite likelihood method. A bootstrap analysis was performed using 1000 replicates.

The neutral community model (NCM) was conducted to assess the potential contribution of stochastic processes in shaping the assembly of comammox bacterial community by modeling and predicting the relationship between OTU relative abundance and their occurrence frequency at different sites along the PRE (57, 58). Model calculations were performed in R using the minpack.lm and HMisc packages. The beta nearest-taxon index (βNTI) was used to evaluate the relative importance of stochastic and deterministic processes using the ses.comdistnt function in the R MicEco package. The dominance of a stochastic processes (dispersal and drift) could be inferred when βNTI values were between -2 and 2. βNTI values less than -2 or greater than 2 suggest that deterministic processes (homogeneous and heterogeneous selection) may be more important (59). RDA was conducted using the CANOCO (version 5.0) to explore the influence of environmental factors on the comammox bacterial community. Permutational multivariate analysis of variance (PERMANOVA) was used to evaluate the differences in comammox community composition among the samples using the adonis function of the vegan package in R. Pearson and Spearman correlations calculated using the corrplot package in R were used to explore the relationships between the abundance of AOA, AOB, comammox, and NOB with sediment physiochemical parameters. Mantel test was conducted to uncover the correlation between the environmental factors and ammonia oxidizer community structure, and the results were combined using the ggcor package in R.

### Data availability.

The high-throughput sequencing raw reads amplified in this study were deposited in the National Omics Data Encyclopedia (NODE) under project ID OEP003197.
