# Supplementary material for: Deterministic Factors Determine the Comammox Community Composition in the Pearl River Estuary Ecosystem
Source: Microbiol Spectr. 2022 Aug 1;10(4):e01016-22. doi: 10.1128/spectrum.01016-22 (PMC9431512; doi:10.1128/spectrum.01016-22)
Supplement: Supplemental file 1 — Tables S1-S3; Fig. S1-S9. Download spectrum.01016-22-s0001.pdf, PDF file, 1.5 MB [file spectrum.01016-22-s0001.pdf]

Deterministic factors determine the comammox community composition in the Pearl River estuary ecosystem

Zongbao Liu [a,b], Qiaoyan Wei [c], Dayu Zou [a], Siyu Zhang [a], Chuanlun Zhang [d], Zhexue Quan [e], Meng Li [a,b*]

[a] *Archaeal Biology Center, Institute for Advanced Study, Shenzhen University, Shenzhen, Guangdong, China.*

[b] *Shenzhen Key Laboratory of Marine Microbiome Engineering, Institute for Advanced Study, Shenzhen University, Shenzhen, Guangdong, China.*

[c] *School of Life and Environmental Science, Guilin University of Electronic Technology, Guilin, Guangxi, PR China*

[d] *Shenzhen Key Laboratory of Marine Archaea Geo-Omics, Southern University of Science and Technology, Shenzhen, Guangdong, PR China*

[e] *Ministry of Education Key Laboratory for Biodiversity Science and Ecological Engineering, Institute of Biodiversity Science, School of Life Sciences, Fudan University, Shanghai, PR China*

***Corresponding author:**

Dr. Meng Li

Mailing address: Institute for Advanced Study, Shenzhen University, Shenzhen, Guangdong, PR China

Tel.: +86-755-26979250

E-mail Address: limeng848@szu.edu.cn

Running title: Comammox community assembly in the PRE ecosystem

Table S1 qPCR Primers used in this study

Target gene	Primer	Sequence (5’-3’)	Length of product (bp)	Annealing temperature (°C)	Reference
16S rRNA	515F	GTGCCAGCMGCCGCGGTAA	290	55	(1)
	806R	GGACTACHVGGGTWTCTAAT			
AOA *amoA*	cren amo_F	ATGGTCTGGCTAAGACGMTGTA	632	55	(2, 3)
	Arch-*amoA*R	GCGGCCATCCATCTGTATGT			
AOB *amoA*	*amoA*-1F	GGGGTTTCTACTGGTGGT	491	58	(4)
	amoA-2R	CCCCTCKGSAAAGCCTTCTTC			
Comammox *amoA*	A414F	TGGTGGTGGTGGTCNAAYTAT	278	57	(5)
	C616R	ATCATCCGRATGTACTCHGG			
Nitrospira nxrB	nxrB169f	TACATGTGGTGGAACA	485	56	(6)
	nxrB638r	CGGTTCTGGTCRATCA			

Table S2 The characteristics of the sediment samples used in this study

Samples[#]	Longitude	Latitude	Depth[+] m	pH*	Salinity* psu	NH_4^+ mgN/kg	NO_2^- mgN/kg	NO_3^- mgN/kg	TN mgN/kg	TOC mgC/kg	TC mgC/kg
PRE1	113.48	23.07	11	7.50	0.3	107.65	0.23	1.43	3247	2431	2535
PRE2	113.65	22.74	11	7.01	10.0	22.55	0.27	1.55	2236	1483	1567
PRE3	113.72	22.63	15	7.79	10.3	61.96	0.26	1.19	3535	2238	2359
PRE4	113.80	22.32	12	7.69	20.8	12.99	0.23	1.70	1046	1054	1128
PRE5	113.74	22.12	10	7.84	26.2	20.43	0.29	1.11	2336	1837	1943
PRE6	113.67	21.97	8	7.92	31.3	23.14	0.37	2.22	2980	1445	1536
PRE7	113.12	21.82	9	7.30	33.0	11.43	0.29	0.84	1557	995	1115
PRE8	113.90	21.99	22	7.67	34.0	8.68	0.33	1.69	1847	846	931
PRE9	113.96	21.86	25	7.70	39.0	9.98	0.30	1.76	1321	584	677
PRE10	113.71	21.64	34	7.43	34.5	20.70	0.35	1.72	2590	1671	1755
PRE11	113.88	21.71	36	7.64	37.0	9.60	0.33	1.46	1607	778	870
PRE12	114.36	21.96	37	7.54	36.0	10.29	0.29	1.48	2085	790	892
DSR3_wet	113.95	22.58	0.4	7.10	0.2	412.48	0.36	2.05	2368	4830	4846
DSR6_wet	113.95	22.54	2	6.98	0.2	192.37	0.26	1.20	917	1851	1869
SZB1_wet	114.01	22.50	0.9	7.15	6.6	50.14	0.21	1.21	1615	3540	3554
SZB5_wet	113.97	22.49	2.5	7.25	11.9	31.48	0.19	0.76	967	1834	1848
SZB10_wet	113.91	22.45	4.2	7.39	10.4	19.28	0.25	0.81	905	2140	2155
DSR3_dry	113.95	22.58	0.3	7.05	0.2	423.77	0.19	1.58	2184	5436	5468
DSR6_dry	113.95	22.54	2	7.30	1.4	63.94	0.12	0.53	477	671	686
SZB1_dry	114.01	22.50	1.4	7.16	17.0	30.61	0.10	0.69	1361	2069	2080
SZB5_dry	113.97	22.49	3.5	7.31	25.7	22.07	0.23	0.66	955	1662	1676
SZB10_dry	113.91	22.45	5.3	7.30	27.2	15.11	0.16	0.71	848	1449	1462

#: PRE: Pearl River estuary; DSR: Dasha river; SZB: Shenzhen Bay; wet: wet season; dry: dry season.

+: Water depth upon the surface sediments.

*: Parameters of pore waters extracted from each sediment.

Table S3 The effects of environmental factors on comammox community.

Values shown in bold are probability with significant results ($P < 0.05$).

Environmental factors	Explains (%)	pseudo-F	*P* value
Salinity	12.7	3.2	**0.002**
TN	12.5	2.9	**0.009**
NH_4^+	9.7	2.7	**0.004**
Depth	7.9	2.3	**0.02**
TOC	4	1.2	0.284
NO_2^-	4.2	1.3	0.254
pH	3.5	1.1	0.364
TC	3	0.9	0.472
NO_3^-	2.7	0.8	0.536

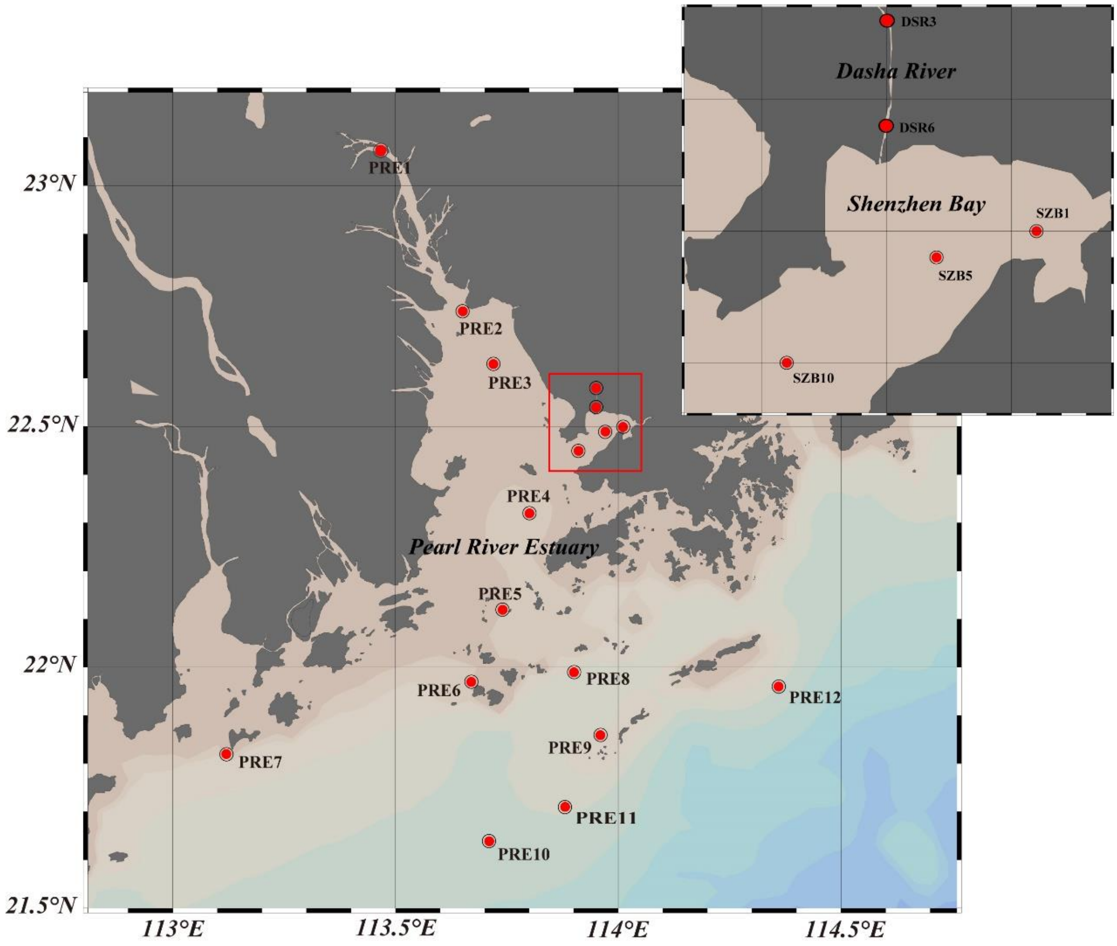

Fig. S1 Sampling stations in the PRE. DSR: Dasha river; SZB: Shenzhen Bay; PRE: Pearl River estuary.

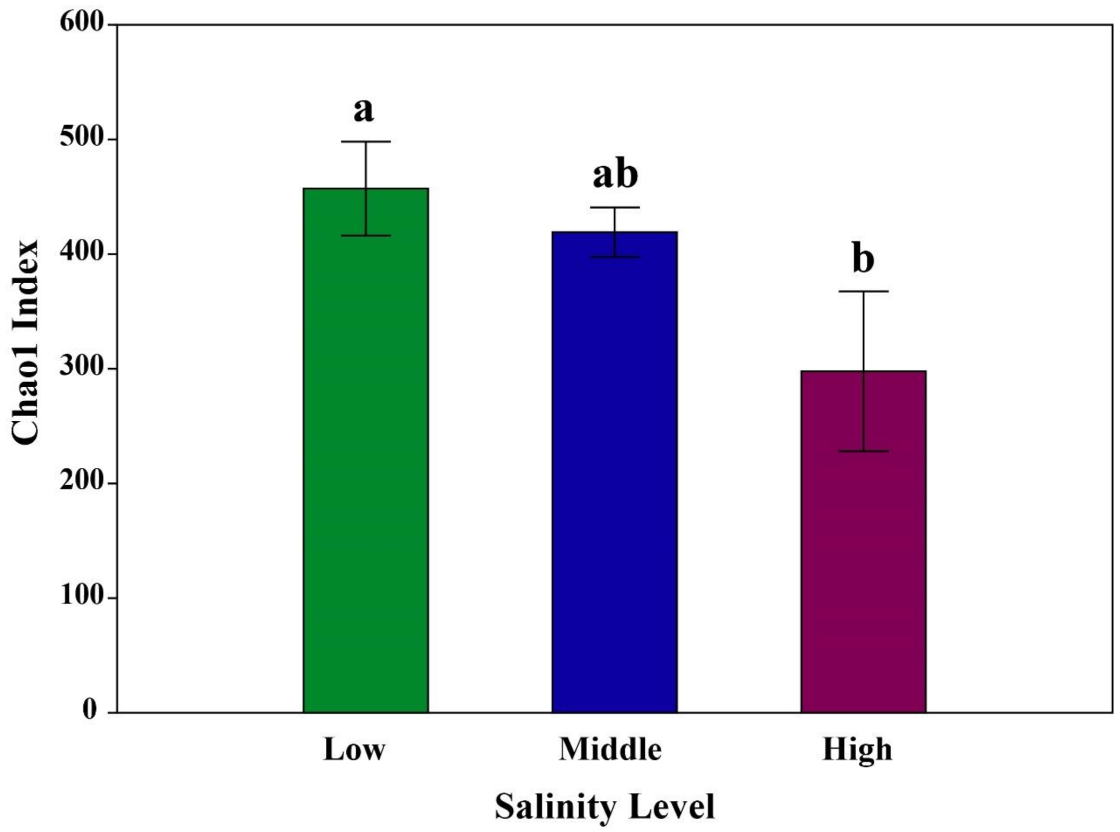

Fig. S2 Boxplots showing the variations in the Chao1 index of comammox community in the PRE sediments with different salinity levels. The lower case letters (a, b) indicate significant differences among different sites (one-way ANOVA; Duncan test; $P < 0.05$).

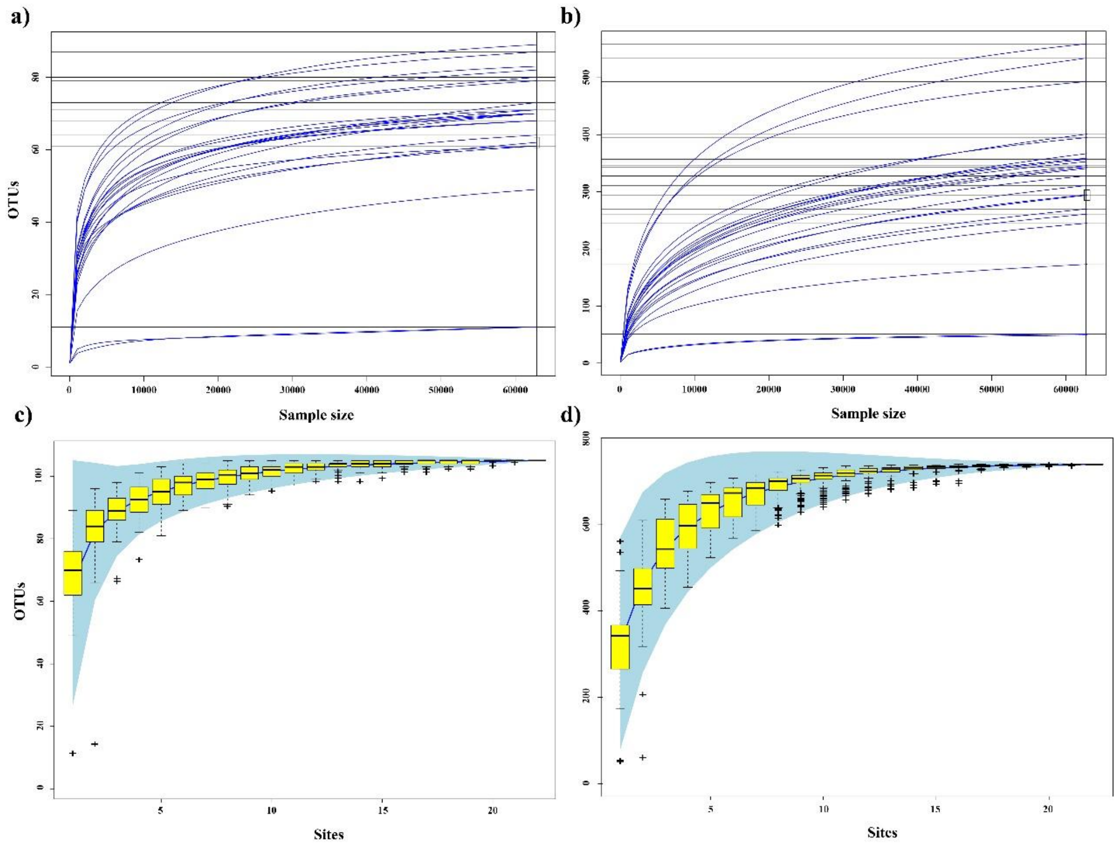

Fig. S3 Species rarefaction curve of the number of OTUs based similarity thresholds of 90% (a) and 95% (b); Species accumulation curve of the number of OTUs based similarity thresholds of 90% (c) and 95% (d)

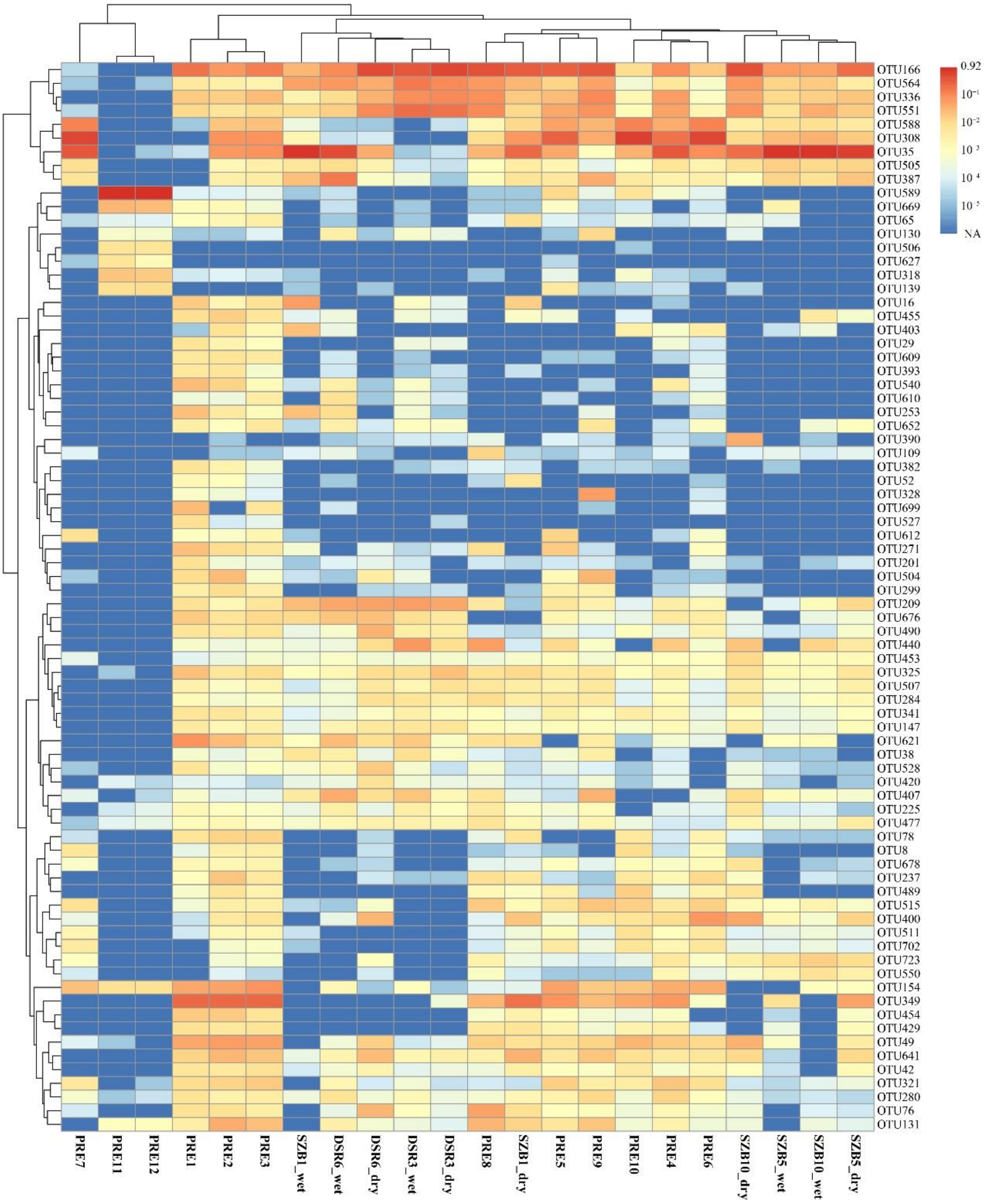

Fig. S4 Heatmap displaying the relative abundances of the 78 major OTUs (relative abundance > 5×10^{-3} ratio in at least one sample) retrieved from the 22 PRE sediments.

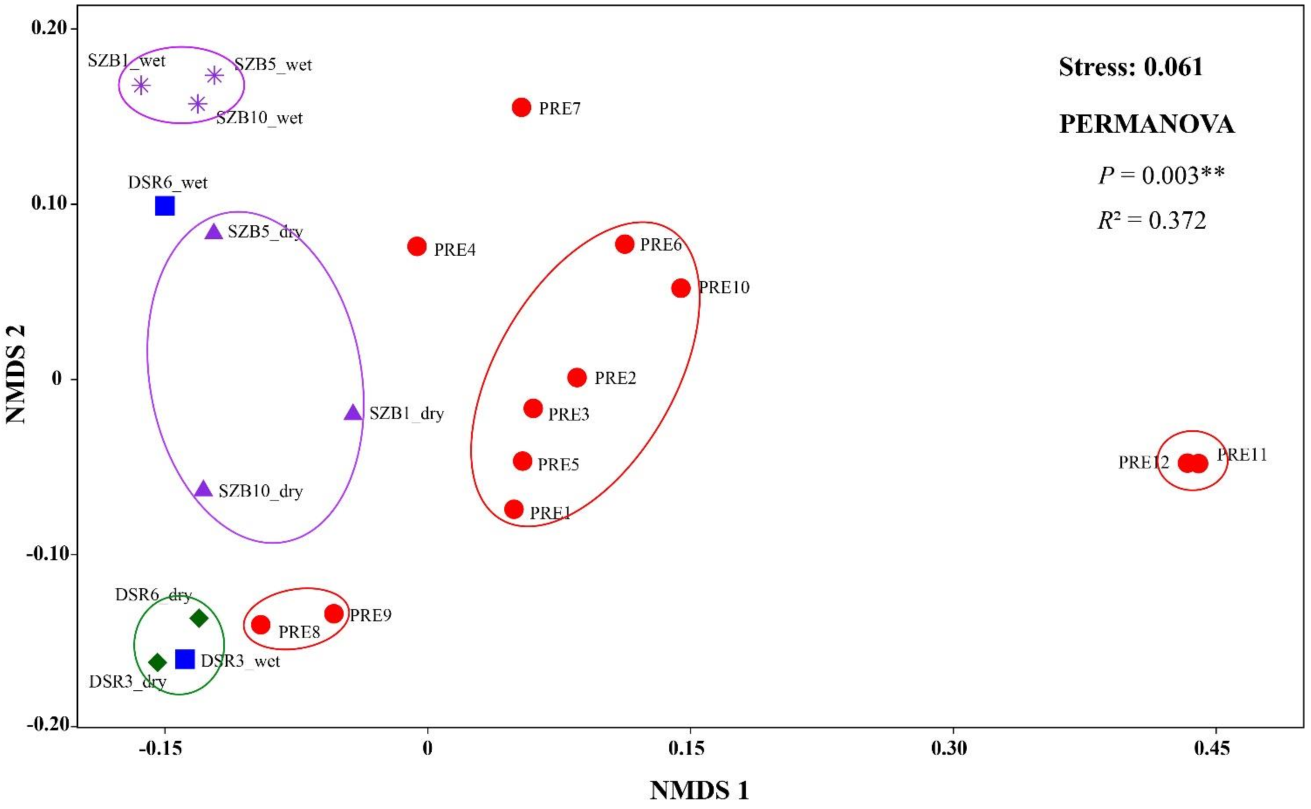

Fig. S5 NMDS plot showing the comammox community composition differences among the 22 PRE sediments.

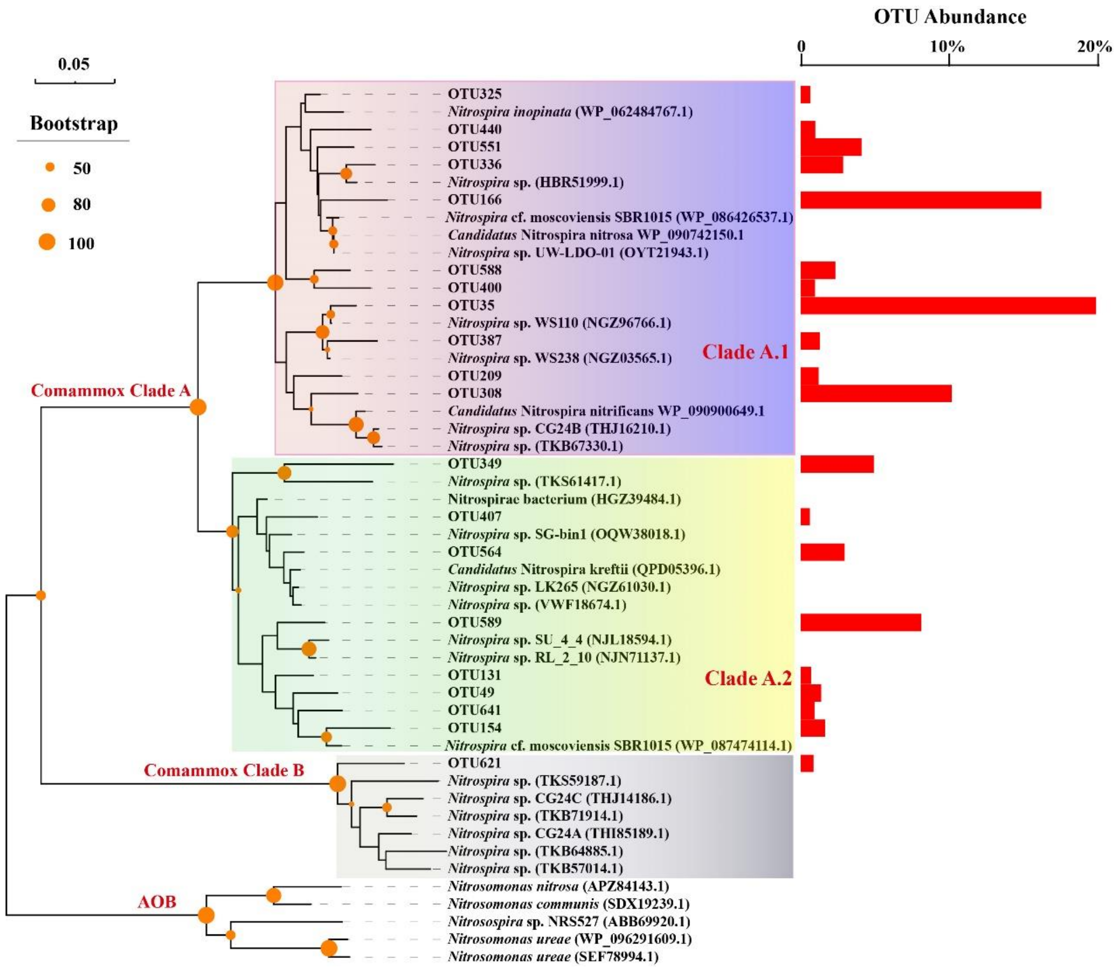

Fig. S6 Neighbor-joining phylogenetic tree of comammox *amoA* based on 20 major OTUs (with proportion higher than 0.5%) with a 95% similarity cutoff. The bar graph indicates the relative proportion of each OTU in the 22 PRE sediments.

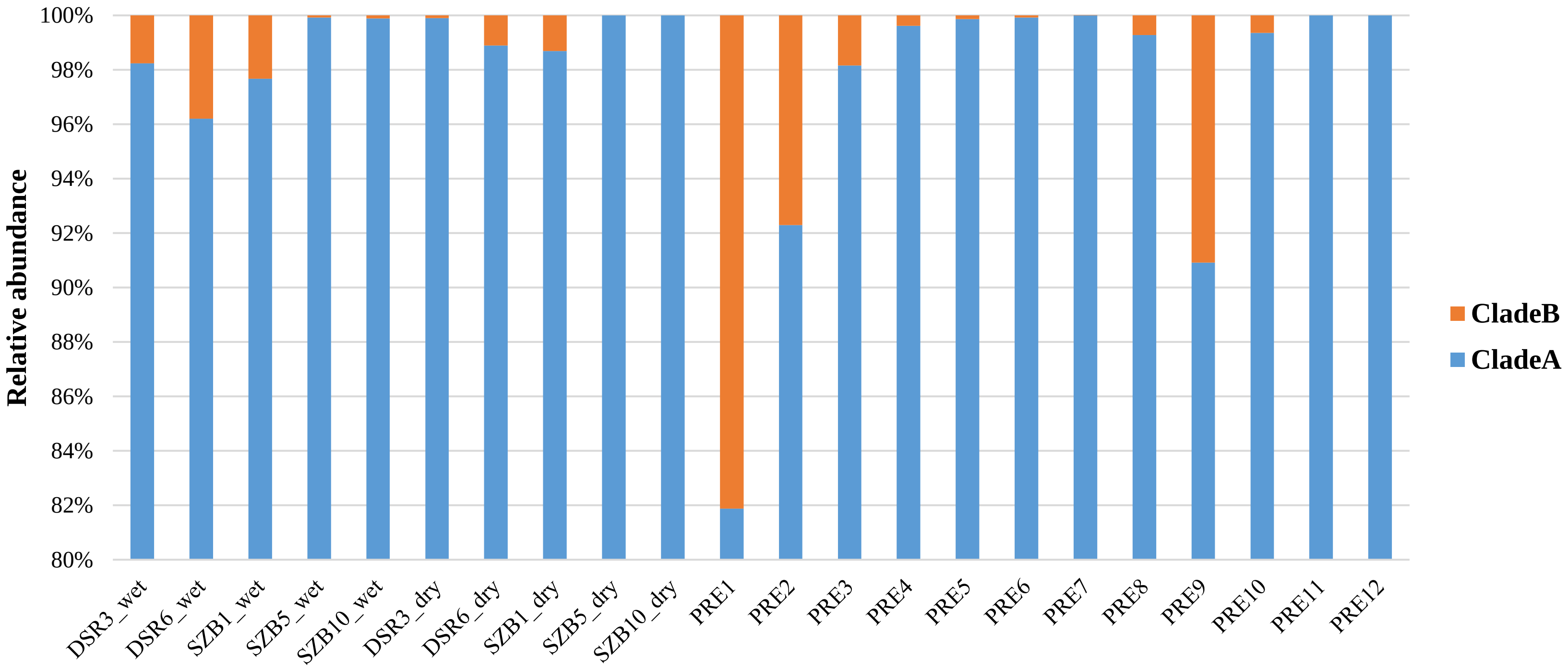

Fig. S7 Relative proportions of the different clades of comammox in the 22 PRE sediments.

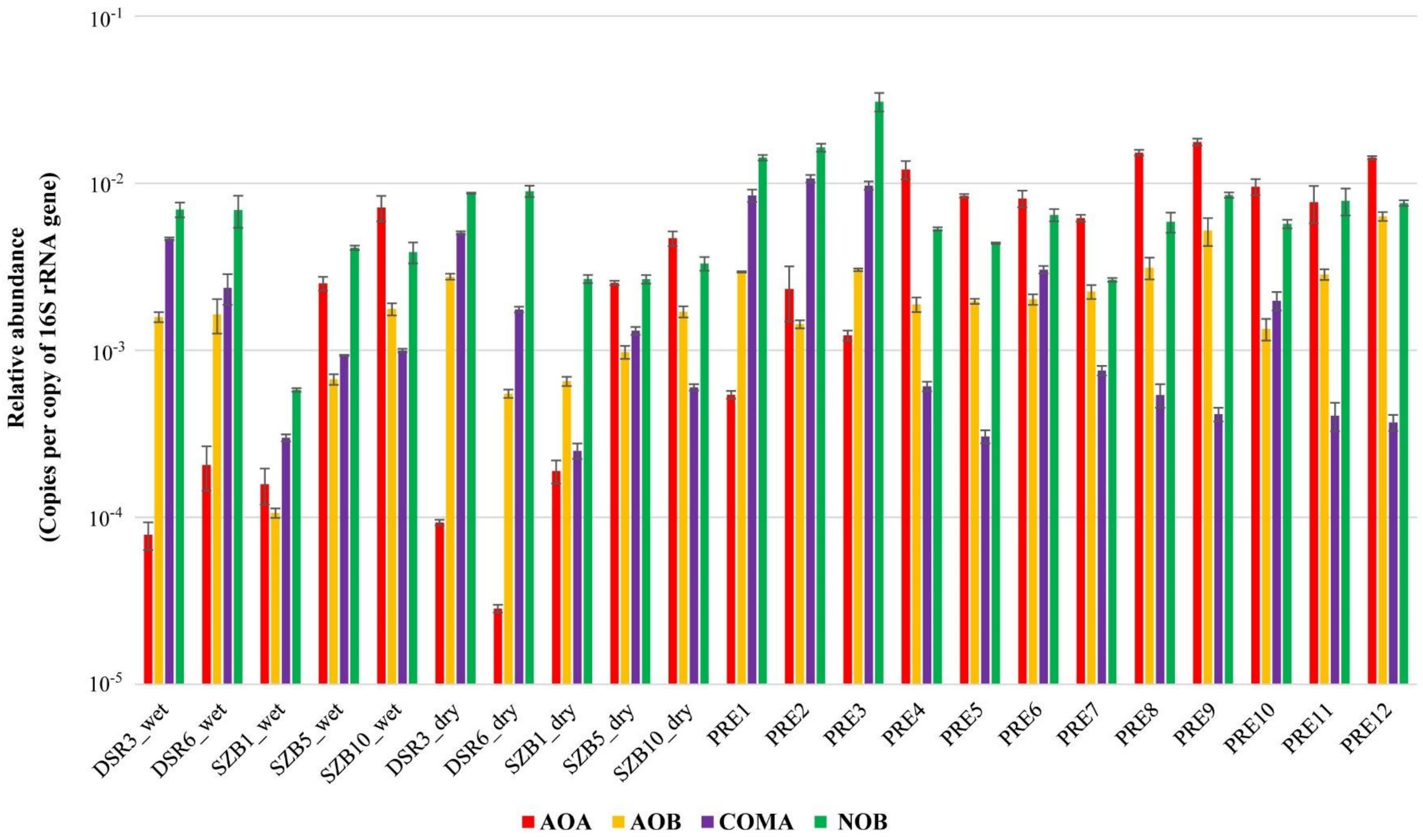

Fig. S8 Relative abundances of AOA, AOB, comammox and NOB in the 22 PRE sediments.

The error bars indicate the standard deviation of three replicates.

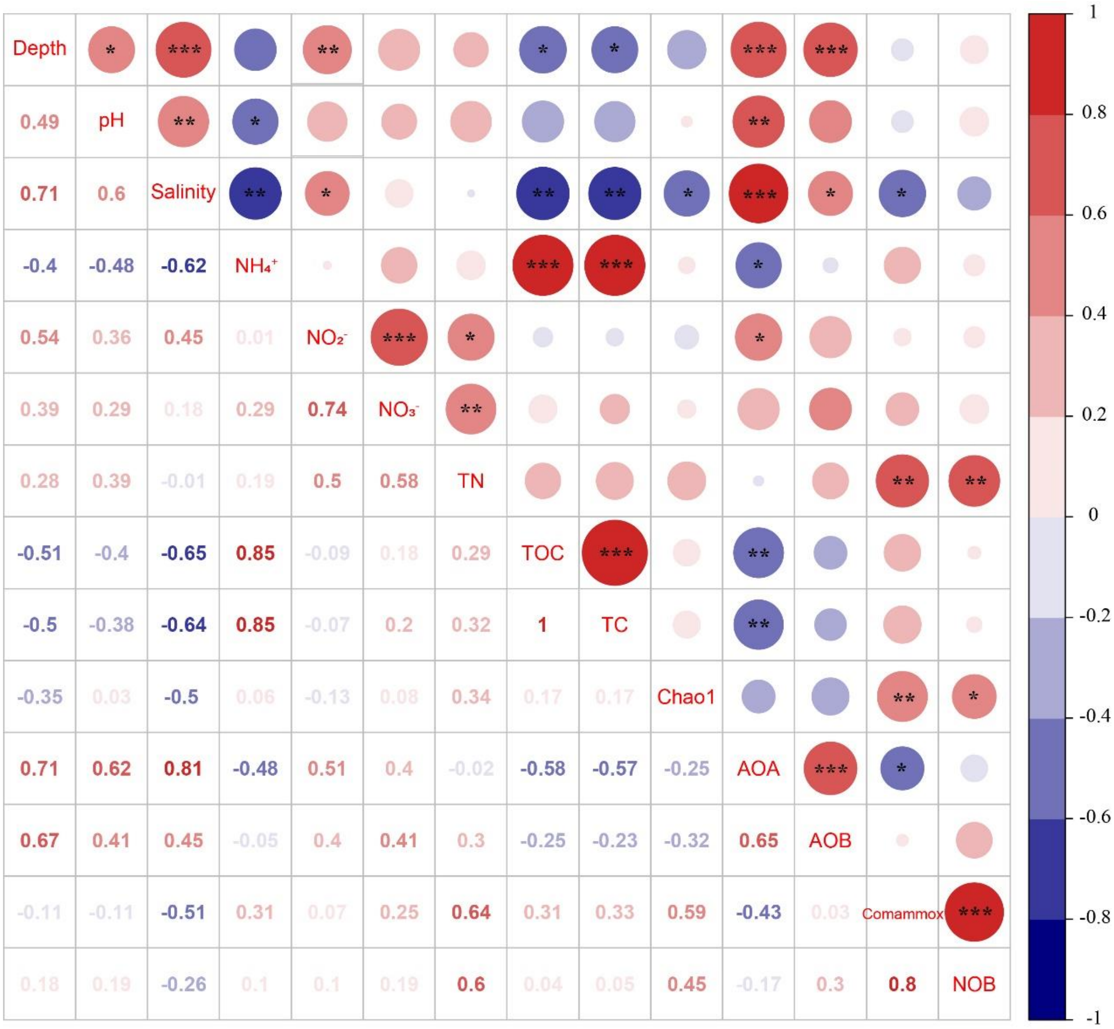

Fig. S9 The Pearson correlation coefficients between environmental factors and comammox Chao1 richness, and relative abundances of AOA, AOB, comammox and NOB. Chao1 index was calculated based on 95% amino acid identity cutoff for comammox *amoA* gene sequences; Red squares, positive correlations; blue squares, negative correlations. *, **, and *** represent the significance at 0.05, 0.01, and 0.001 level, respectively.

References

1. Caporaso JG, Lauber CL, Walters WA, Berg-Lyons D, Lozupone CA, Turnbaugh PJ, Fierer N, Knight R. 2011. Global patterns of 16S rRNA diversity at a depth of millions of sequences per sample. PNAS 108:4516–4522.
2. Francis CA, Roberts KJ, Beman JM, Santoro AE, Oakley BB. 2005. Ubiquity and diversity of ammonia-oxidizing archaea in water columns and sediments of the ocean. PNAS 102:14683-14688.
3. Hallam SJ, Mincer TJ, Schleper C, Preston CM, Roberts K, Richardson PM, DeLong EF. 2006. Pathways of carbon assimilation and ammonia oxidation suggested by environmental genomic analyses of marine Crenarchaeota. PLoS Biol. 4:e95.
4. Rotthauwe JH, Witzel KP, Liesack W. 1997. The ammonia monooxygenase structural gene amoA as a functional marker: molecular fine-scale analysis of natural ammonia-oxidizing populations. Appl Environ Microbiol 63:4704-4712.
5. Xia F, Wang G, Zhu T, Zou B, Rhee SK, Quan X. 2018. Ubiquity and diversity of complete ammonia oxidizers (comammox). Appl Environ Microbiol 84:e01390-01318.
6. Pester M, Maixner F, Berry D, Rattei T, Koch H, Lücker S, Nowka B, Richter A, Spieck E, Lebedeva E, Loy A, Wagner M, Daims H. 2014. NxrB encoding the beta subunit of nitrite oxidoreductase as functional and phylogenetic marker for nitrite-oxidizing N itrospira. Environ Microbiol 16:3055-3071.
